# Solitary Osteochondroma of the Ventral Scapula Associated with Large Bursa Formation and Pseudowinging of the Scapula: A Case Report and Literature Review

**DOI:** 10.1155/2018/5145642

**Published:** 2018-02-05

**Authors:** Kiyohisa Ogawa, Wataru Inokuchi

**Affiliations:** Department of Orthopedic Surgery, Eiju General Hospital, 2-3-23 Higashiueno, Taito-ku, Tokyo 110-8645, Japan

## Abstract

Osteochondroma (OC) is the most common benign bone tumor and may occur on any bone in which endochondral ossification develops. Although scapular OC accounts for less than 5% of the cases of solitary OC, OC is the most common lesion among the tumors and tumor-like lesions of the scapula. OC that develops near the medial scapular border easily causes friction with the ribcage; hence, almost half the number of cases of OC associated with marked bursa formation develops in the ventral scapula. We report a case of a 27-year-old female with a painful OC of the ventral scapular surface associated with large bursa formation and pseudowinging of the scapula. After l2 years of follow-up with magnetic resonance imaging, we confirm that the accompanied bursa left at surgery disappears.

## 1. Introduction

Osteochondroma (OC) is the most common benign bone tumor, of which the detailed development process is still debatable. The reported incidence of OC is 33–35% of benign and 8–10% of all surgically removed bone tumors, which may be an underestimation as most OCs are asymptomatic and are never found [1, 2]. OC may occur on any bone in which endochondral ossification develops. The most common sites of OC development are the metaphyseal region of the long bones of the limbs, while flat bones are less commonly involved [2]. Symptoms often develop in relation to the size and location of the OC lesion. Pain may result from fracture, bursa formation, arthritis, and impingement of adjacent tendons, blood vessels, nerves, or the spinal cord [1, 2]. We describe a case of a 27-year-old female with a painful OC on the ventral surface of the scapula associated with a large bursa and pseudowinging of the scapula with l2 years of follow-up. Written informed consent was obtained from the patient for publication of this case report and accompanying images.

## 2. Case Report

A 27-year-old right-hand-dominant and otherwise healthy female student presented with a pain in the right upper scapular region that increased with shoulder motion and resting in the supine position. She reported that a painless snap in her back had occurred during sports activity 11 years previously and had disappeared 2 years later after discontinuing sports activity. The pain in the right upper scapular region had appeared 3 months earlier during continuous inputting to her personal computer and had rapidly worsened. She presented at a nearby hospital because the pain was generated during activities of daily life and prevented her from sleeping in the supine position. A physician suspected a malignant bone tumor based on radiographic and magnetic resonance imaging (MRI) findings and referred the patient to our hospital.

**Figure 1:**
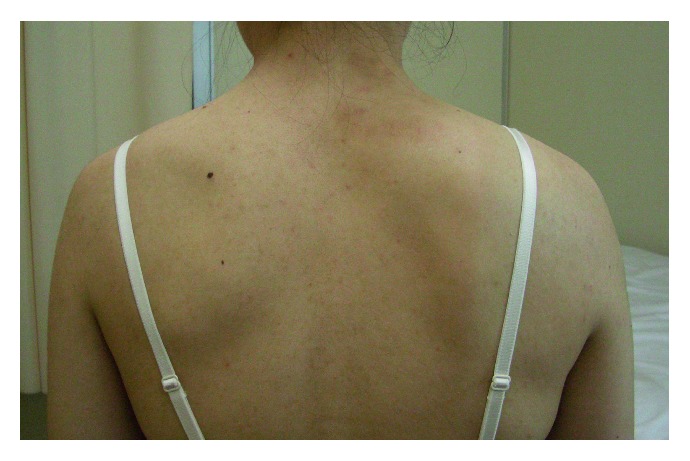
Preoperative photograph showing the deformity of the right scapula. The right scapula showed winging with the arm at the side. An upper interval between the spine and medial scapular border was widened, but the lower one was not.

**Figure 2:**
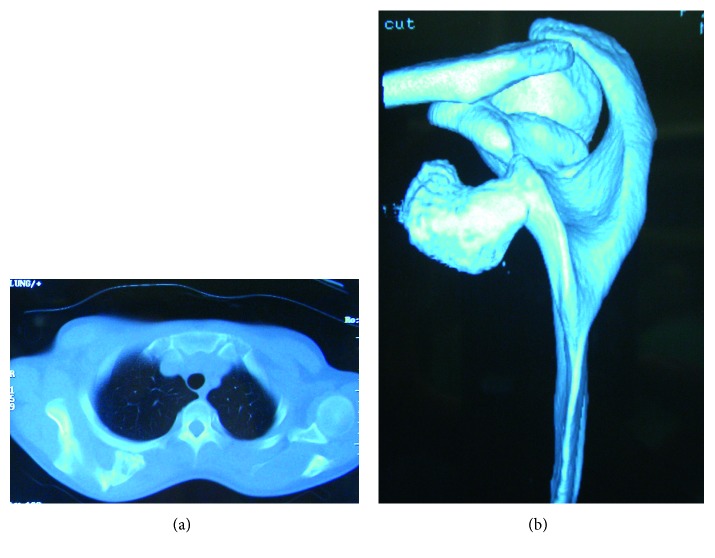
Preoperative computed tomography (CT) findings. (a) CT showed the mushroom-shaped osseous tumor composed of a cortex continuous with the scapular cortex, with a broad flat distal end that almost contacted the third rib. (b) Three-dimensional CT revealed the close relationship between the base of the osseous tumor and the superomedial scapular angle.

**Figure 3:**
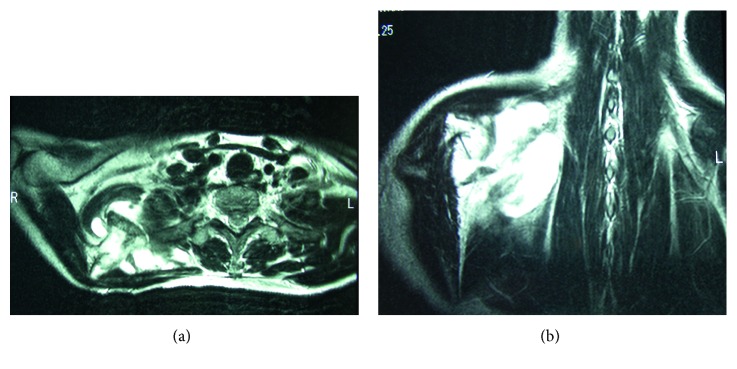
Preoperative T2-weighted magnetic resonance imaging. (a) Axial view demonstrated the cystic lesion containing a large amount of fluid that surrounded the osseous tumor. (b) Coronal view revealed that the cystic lesion spread over the upper two-thirds of the scapula.

The patient had no relevant family or medical history. The right shoulder was slightly lower than the contralateral shoulder. There were no neurological deficits in the right shoulder or arm. There was winging of the right scapula with the arm at the side. An upper interval between the spine and the medial scapular border was widened by 70%, but the lower one was not (Figure 1). There was no atrophy of the back muscle, and contraction of the trapezius was normal. The muscle bellies of the short rotators and rotator cuff were not tender and were without defects. The limitations of the active ranges of motion were 10° for total elevation, 15° for external rotation, and two vertebrae for internal rotation. Horizontal adduction was not limited with moderate pain beyond 100°. The empty can test generated upper scapular pain. No deformity or osseous tumor was palpable in the large joints, pelvis, or ribcage. Radiographs revealed a bone mass extruding from the ventral side of the superomedial scapular angle; no abnormality was depicted in the large joints. Computed tomography showed a mushroom-shaped osseous tumor composed of a cortex continuous with the scapular cortex, with a broad flat distal end that almost contacted with the third rib, as well as a soft tissue tumor between the serratus anterior and the ribcage (Figures 2 and 2). MRI taken at the previous hospital revealed a cystic lesion containing a large amount of fluid that surrounded the osseous tumor and spread over the upper two-thirds of the scapula (Figures 3 and 3). Marked rim enhancement was demonstrated on contrast-enhanced T1-weighted imagery with fat suppression. From these imaging findings, our diagnosis was a solitary OC with a large bursa; the possibility of malignant transformation was considered to be low.

**Figure 4:**
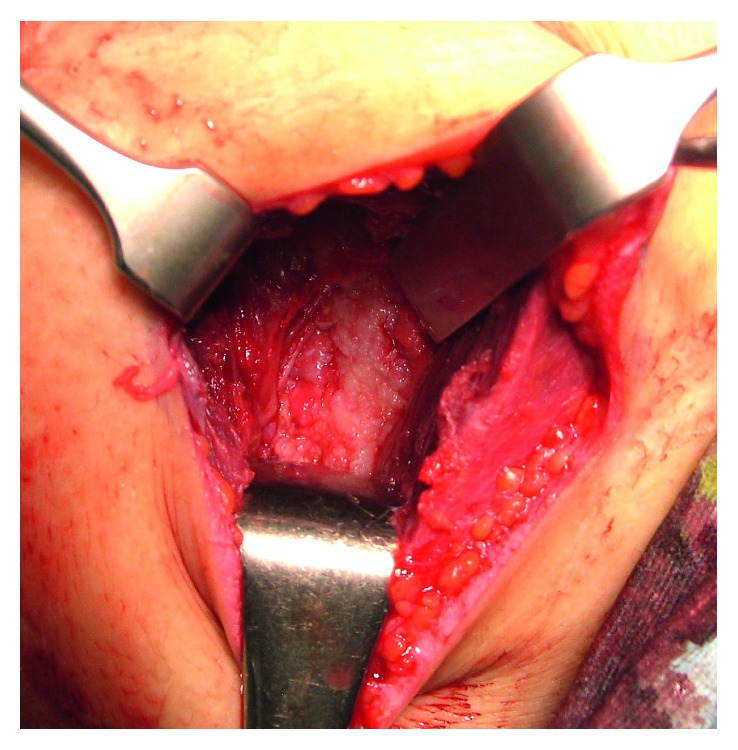
Intraoperative photograph showing the exposure of the rib. After resection of the osseous lesion, a large cystic space appeared, covered by a white thick membrane; at the bottom of this space, a 1 × 3 cm section of rib surface covered by edematous synovium was exposed in a defect of the thick membrane.

We performed surgery 3 months after the patient's first visit to our hospital. Under general anesthesia, the patient was placed in the lateral decubitus position with the shoulder flexed and abducted at 110°. The upper extremity was supported on a pillow to avoid excessive horizontal adduction so that the superomedial scapular angle was situated just underneath the middle trapezius. A 10 cm longitudinal incision was made along and 3 cm medial to the medial scapular border, centering at the most proximal end of the scapular spine. The trapezius and rhomboid minor were divided along their fibers. The thick and edematous cyst wall exposed in the bottom of the operative field was cut, and about 100 ml of clear yellow-brown fluid flowed out. The stalk of the OC arose in the superomedial scapular angle 1 cm lateral to the medial scapular border and penetrated the subscapularis and serratus anterior muscles. The base of the lesion was cut with its periosteum along the ventral scapular surface. We cut the attached thick fibrous tissue attached around the distal stalk, which was considered to be a bursal wall. After resection of the osseous lesion, a large cystic space appeared, covered by a white thick membrane considered to be inflamed synovial tissue; at the bottom of this space a 1 × 3 cm section of the rib surface was exposed in the defect of the thick membrane (Figure 4). There was no free body in the space or palpable indurations on its wall. We irrigated the space, sutured the fascias of the divided muscles, and subsequently closed the skin.

**Figure 5:**
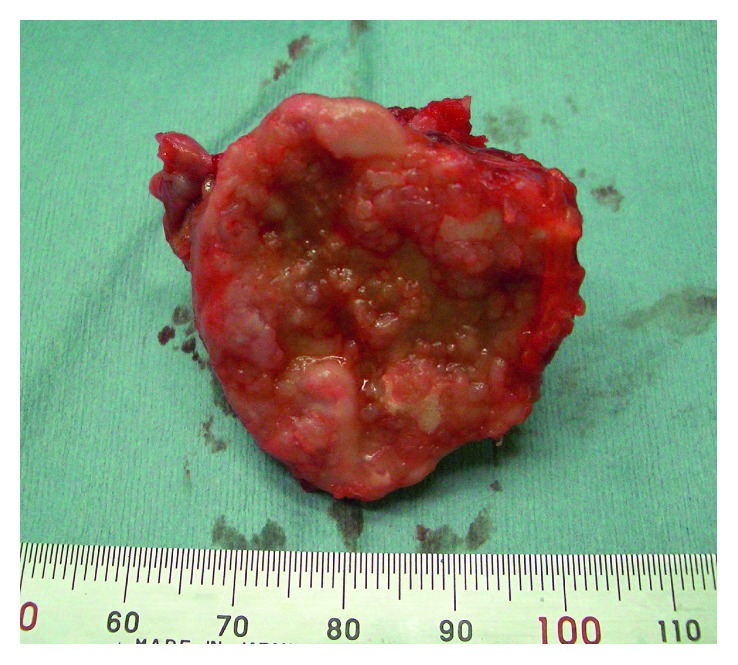
Macroscopic findings of the distal end of the osseous tumor. The distal surface was rather irregular. The typical thick perichondrium and cartilage cap were not found, although it was covered by spotty fibrocartilage-like tissue.

Macroscopically, the typical thick perichondrium and cartilage cap were not found, although the flat distal end of the lesion was covered by spotty fibrocartilage-like tissue (Figure 5). Histological examination revealed characteristic findings of OC without any malignant changes. The distal end was covered by synovial tissue and was composed of bony trabeculae in which mostly fatty tissue and bone marrow intervened.

**Figure 6:**
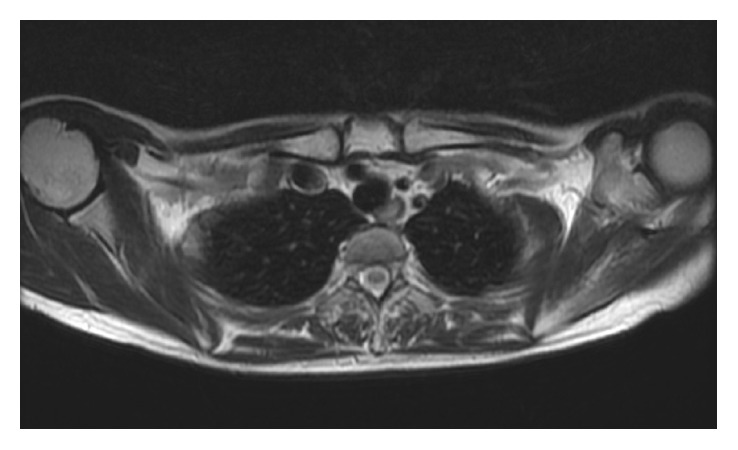
T2-weighted magnetic resonance images taken at the final follow-up 12 years postoperatively. There was no abnormality of the soft tissue or bony structures, although some asymmetry existed.

The postoperative course was uneventful. The patient returned to normal daily life and full activity 3 weeks postoperatively. At the time of final follow-up 12 years postoperatively, the right scapula was in the normal position, the scapulothoracic rhythm was symmetrical, and there was no limitation of active range of motion or any associated crepitus. The Constant score ratio compared with the contralateral left shoulder was 100% [3]. MRI showed no abnormality of the soft tissue or bony structures (Figure 6).

## 3. Discussion

OC is the most common benign bone tumor [1, 2]. It has been reported that 14% of OC are hereditary multiple OC (an autosomal-dominant disorder) and 86% are solitary OC without heredity [1, 4]. Solitary OC is a common lesion estimated to occur in 1-2% of individuals [4]. The literature indicates little sex predilection [1]. The cartilaginous cap is the site of active growth, and the degree of maturity parallels the host bone. If the OC is arrested, as may occur in adults, there may be practically no cartilaginous cap. This appearance is especially likely with the rare OC associated with an overlying bursa [1]. In a minority of cases, OC may grow beyond skeletal maturity [5]. The lesion in our case was a solitary OC, as the physiological and radiographic investigations found no other bony abnormalities. The OC was considered to be arrested, as there was no cartilaginous cap histologically.

The most common sites of involvement are the metaphyseal region of the long bones of the limbs, while involvement of flat bones is less common [2]. The scapula is reportedly the site of occurrence of 1–3% of all primary bone tumors [6]. OC is the most common of the tumors and tumor-like lesions of the scapula, accounting for 17–40% [6, 7]. The majority of scapular tumors are of cartilaginous origin; this may be because the scapula develops by endochondral ossification from seven ossification centers and has a total length of physes exceeding any tubular bone [6]. Scapular lesions account for 4–4.9% of the solitary OC cases [1, 4].

The vast majority of OC are asymptomatic. Symptoms are often related to the size and location of the lesion [2]. The most common symptom is a nontender, painless cosmetic deformity related to the slowly enlarging mass. Additional complications that cause pain include fracture, bursa formation, arthritis, and impingement of the adjacent tendons, blood vessels, nerves, or spinal cord [1, 2, 4]. Approximately 60–80% of patients with OC were younger than 20 years at the time of the first excision of the OC [1, 4]. Although it is uncommon for a solitary OC to become symptomatic after skeletal maturity, there are some well-documented reports of the appearance of symptoms in adults that resulted from the following: mechanical irritation of muscle, tendon, or soft tissue; compression of a nerve; formation of a pseudoaneurysm or an adventitious bursa; fracture; infection; ischemic necrosis; or malignant transformation [8]. In our case, the symptoms appeared after skeletal maturity.

In 1891, Orlow [9] originally described the bursa formation between an OC and the surrounding soft tissue as “exostosis bursata,” although this had been recognized previously. The exact occurrence rate of bursal formation associated with OC is unknown, but Unni and Inwards [1] stated that an overlying bursa was significant enough to be described in 1.3% of their cases treated surgically. McWilliams [10] reported the first case of ventral-side scapular OC accompanied by adventitious bursa. Since this first reported case, to the best of our knowledge, there have been 20 cases of the solitary ventral-side scapular OC associated with a large bursa (including our case) reported in the English literature (Table 1). The scapula glides over the thoracic wall, cushioned from the undulating surface of the ribs by the serratus anterior and subscapularis muscles. However, the scapular superior and inferior angles, and its medial border, are relatively poorly cushioned [11]. Endochondral ossification occurs at the medial border and lateral angle of the scapula, where OC commonly develops. OC near the medial scapular border then easily causes friction with the ribcage. In the literature, 41–73% of OC associated with marked bursa formation are located on the ventral scapula [4, 12–14]. Many authors described that the scapular OC with a large bursa penetrated the subscapularis and serratus anterior muscles and that the bursae were formed along the chest wall; although Chiarelli et al. reported an OC case associated with intramuscular bursa formation in the serratus anterior [15]. It remains unknown whether the bursa associated with the OC in our case was an adventitious bursa or an inflamed consistent bursa located between the serratus anterior and the ribcage [16]. These bursae associated with OC are lined by synovium and may hemorrhage or become inflamed or infected. In addition, the bursa may contain chondral or fibrin bodies, and chondrometaplasia can occur within the synovial lining, leading to secondary synovial chondromatosis [4, 14, 17–20]. In our case, there was no free body in the bursa and no palpable induration on the bursal wall that would have indicated chondrometaplasia or secondary osteochondromatosis. Traces of direct contact between the lesion and the ribs were apparent, and the bursa was formed between the serratus anterior and the chest wall. These findings are consistent with other reports that described the existence of resorption or erosion of the contacting ribs [21, 22].

Scapular winging and snapping have been well recognized as symptoms of OC development on the ventral scapula. The causes of scapular winging are numerous. Increased scapular prominence from causes other than paralysis of the serratus anterior muscle is referred to as “pseudowinging” of the scapula [23]; this pseudowinging often leads to the discovery of OC on the ventral scapula. Of the 20 reported cases of ventral scapular OC (including our case), 14 cases showed pseudowinging (Table 1). The types of winging indicate the developing location of the OC, although they are characteristically present even at rest with the arm at the side. Carlson et al. [24] analyzed the etiologies of the snapping scapula syndrome causing shoulder discomfort characterized by painful, audible, and/or palpable abnormal scapulothoracic motion in 89 reported cases. They found that the most common causes of snapping scapula syndrome were skeletal abnormalities (43% of reported cases), of which three were OC. Of 20 reported cases of ventral scapular OC (including our case), 14 cases had symptoms consistent with snapping scapula syndrome (Table 1). Therefore, snapping scapula is a common symptom in cases of ventral scapular OC, although OC is an uncommon cause of snapping scapula.

Malignant transformation of OC is well known. WHO reported that the risk of malignant transformation to secondary peripheral chondrosarcoma is estimated at about 1% for solitary and up to 5% for multiple OC [2]. Some authors reported that 27.3–36.3% of patients with multiple OC who underwent surgery had secondary chondrosarcoma, whereas only 3.2–7.6% of patients with the solitary form had malignant changes [1, 25, 26]. Nevertheless, follow-up studies in a number of patients with multiple hereditary OC revealed that malignant change occurs in less than 1% of patients [27]. Secondary chondrosarcoma in OC reportedly has a predilection for flat bones [26]. Clinically, malignant transformation should be suspected in lesions that grow or cause pain after skeletal maturity, as OC only rarely enlarges after this time [4]. The most frequent imaging findings of malignant transformation are irregularity or indistinctness of the surface of the OC, areas of lucency and inhomogeneous mineralization within the OC, and a soft tissue mass frequently containing foci of scattered, punctuate calcifications [26]. On MRI, malignant transformation should be suspected in large tumors with cartilaginous caps that are thick (>1.5–2 cm in an adult) and unmineralized [2]. However, differentiation between the fluid in the inflamed bursa and a large cartilaginous cap is difficult, as they have similar signal characteristics on MRI [5]. In our case, there were no characteristic radiographic findings that indicated malignant transformation. The existence of a thick cartilaginous cap was almost definitively ruled out preoperatively, as the interval between the distal end of the OC and the opposite ribs was 2-3 mm. Histopathological examination confirmed that there were no malignant findings and no cartilaginous cap.

In the approach for OC on the ventral side of the superomedial scapular angle, the superior fibers of the trapezius must be separated. This approach carries a risk of harm to the spinal accessory nerve, which travels an average of 2.7 cm lateral to the superomedial scapular angle [16]. To minimize this risk, we placed the patient in the lateral decubitus position with the shoulder flexed and abducted at 110°, so that the superomedial scapular angle was situated just underneath the middle fibers of the trapezius. An operative field large enough to remove the OC was gained, but it was impossible to observe the whole interior of the associated bursa. We left the bursa untouched to avoid damaging the long thoracic and dorsal scapular nerves that travel closely along the bursal wall. Of the 19 previously reported cases of ventral scapular OC, complete resection of the bursa was performed in eight cases and partial resection was done in two. Regarding treatment of the bursa, there are two different opinions: one that total resection has to be performed [13] and one that aggressive resection of the bursa is unnecessary as the bursa should not recur once there is no longer irritation of the underlying ribs [22]. Some authors have reported success with no or partial resection of the bursa [22, 28, 29]. The bursa accompanying OC involves inflammation caused by mechanical irritation, which produces excess synovial fluid. When the mechanical irritation disappears, the inflammation resolves and the bursal tissue should be normalized. In our case, MRI taken at the time of final follow-up 12 years postoperatively showed no abnormality of soft tissue. Therefore, the bursa itself may resolve when there is no osteochondral free body and indurations in the bursal wall. Arthroscopic excision recently developed for symptomatic OC is effective if the OC is definitely benign [29].

## Figures and Tables

**Table 1:** Reported cases of solitary ventral-side scapular osteochondroma associated with a large bursa.

Year	Author(s)	Patient's age/sex/affected side^∗^	Duration of symptoms	Scapular winging	Osteochondroma location
1914	McWilliams	18/F/L	1 year	Yes	Lower: inner
1973	Parsons	20/M/L	?	Yes	Vertebral: center
1979	El-Khoury and Bassett	23/M/R	2 months	?	Axillar: midportion
1981	Borges et al.	56/F/L	?	?	Vertebral: 3rd and 4th ribs
1991	Griffiths et al.	38/M/L	?	Yes	Near the inferior angle
1997	Jacobi et al.	17/F/R	7 weeks after injury	?	Near the inferior angle
1999	Okada et al.	33/M/R	1 month	?	Lateral: inferior
2000	Shackcloth and Page	32/M/R	6 months	?	Lateral: center
2006	Mohsen et al.	19/M/R	6 months	Yes	Vertebral: midportion
2010	Scott and Alexander	14/M/R	9 months	Yes	Near the inferior angle
2010	Aalderink and Wolf	Mid-20s/F/R	15 years	Yes	Medial: inferior
2010	Frost et al.	11/F/R	?	Yes	?
		20/M/L	?	Yes	?
		25/M/L	?	Yes	?
2012	Orth et al.	35/F/R	2-3 months	Yes	Near the superior angle
		48/F/L	2-3 years	Yes	Lateral: inferior
2014	Sivananda et al.	31/F/R	6 months	Yes	Superior angle
2015	Flugstad et al.	20/M/L	4 months	Yes	?
2016	Mohamed et al.	30/M/R	1 year		Near the inferior angle
	This study	27/F/R	3 months	Yes	Superior angle

^∗^Patients' ages are given in years. “?” indicates that the information was not provided in the case report.
